# Effectiveness of programme approaches to improve the coverage of maternal nutrition interventions in South Asia

**DOI:** 10.1111/mcn.12699

**Published:** 2018-11-29

**Authors:** Sophie Goudet, Zivai Murira, Harriet Torlesse, Jennifer Hatchard, Jennifer Busch‐Hallen

**Affiliations:** ^1^ School of Sport, Exercise and Health Sciences Loughborough University Loughborough UK; ^2^ Nutrition Section UNICEF Regional Office for South Asia Kathmandu Nepal; ^3^ Global Technical Services Nutrition International Ottawa Canada

**Keywords:** calcium supplementation, counselling, iron folic acid supplementation, maternal nutrition, pregnancy, South Asia

## Abstract

The nutritional status of women before pregnancy, during pregnancy, and after delivery has far reaching consequences for maternal health and child survival, growth, and development. In South Asia, the high prevalence of short stature, thinness, and anaemia among women of reproductive age underlie the high prevalence of child undernutrition in the region, whereas overweight and obesity are rising concerns. A systematic review of evidence (2000–2017) was conducted to identify barriers and programme approaches to improving the coverage of maternal nutrition interventions in the region. The search strategy used 13 electronic bibliographic databases and 14 websites of development and technical agencies and identified 2,247 citations. Nine studies conducted in Bangladesh (*n* = 2), India (*n* = 5), Nepal (*n* = 1), and Pakistan (n = 1) were selected for the review, and outcomes included the receipt and consumption of iron and folic acid and calcium supplements and the receipt of information on dietary intake during pregnancy. The studies indicate that a range of barriers acting at the individual (maternal), household, and health service delivery levels affects intervention coverage during pregnancy. Programme approaches that were effective in improving intervention coverage addressed barriers at multiple levels and had several common features: use of formative research and client assessments to inform the design of programme approaches and actions; community‐based delivery platforms to increase access to services; engagement of family members, as well as pregnant women, in influencing behavioural change; actions to improve the capacity, supervision, monitoring, and motivation of front‐line service providers to provide information and counselling; and access to free supplements.

**Key messages**

- A range of barriers acting at the individual (women's education, knowledge, and decision‐making authority), household (wealth and family support to women), and health service delivery level (access to services, quality of counselling, and supply of supplements) affects the coverage of maternal nutrition interventions during pregnancy in South Asian countries.
- Programme approaches that increased intervention coverage addressed multiple barriers; were informed by formative research; used community‐based delivery platforms to reach women and influential family members with interventions; increased the capacity, supervision, and motivation of front‐line service providers; and provided free micronutrient supplements.
- The body of recent evidence on what works to improve the coverage of maternal nutrition interventions in South Asia is very small. Implementation research and programme evaluations are needed for a better understanding of barriers and enablers and pathways to enhance maternal nutrition intervention coverage across diverse contexts in the region.

## 1 INTRODUCTION

The nutritional status of women before pregnancy, during pregnancy, and after delivery affects their well‐being and has long‐lasting impacts on the survival, growth, and development of children across the first 1,000 days between conception and a child's second birthday and beyond (Black et al., 2013). Women who are short, thin, anaemic, or gain inadequate weight during pregnancy are more likely to suffer adverse birth outcomes, including low birth weight and preterm delivery (Christian et al., 2013; Liu et al., 2017). Severe anaemia and calcium deficiency increase the risk of postpartum haemorrhage and hypertensive disorders, respectively (Rahman et al., 2016; World Health Organization [WHO], 2013), two leading causes of maternal death globally (Say et al., 2014). Despite this compelling evidence, the performance of maternal nutrition programmes in low‐ and middle‐income countries is generally poor. For example, coverage data from 59 low‐ and middle‐income countries indicate that the mean proportion of women who consume iron folic acid (IFA) supplements for at least 90 days during pregnancy is only 31% (Development Initiatives, 2017).

Comprehensive maternal nutrition interventions can promote the health of pregnant women and contribute to healthy fetal growth and development (Bhutta et al., 2013; Dewey, 2016). In November 2016, the WHO released guidelines on improving antenatal care (ANC) for women, including a comprehensive set of recommendations on nutrition interventions during pregnancy (WHO, 2016). Although these new guidelines provide an opportunity to reignite attention on maternal nutrition, concerted efforts are needed to ensure that policy and programme actions translate to nutrition services that reach women with quality and equitable coverage. Women in low‐ and middle‐income countries face substantial barriers to accessing nutrition interventions and adopting recommended nutrition behaviours, including low education and knowledge, lack of family support, and inadequate coverage and quality of maternal health and nutrition services (Kavle & Landry, 2017; Victora et al., 2012).

In South Asia, there has been progress on improving women's nutrition. Our review of data from national demographic, health, and nutrition surveys conducted in the last 15 years indicate that low stature (<145 cm) in women of reproductive age has declined by at least 20% in three out of four countries with available data, and low body mass index (BMI; <18.5 kg/m^2^) has fallen by at least 30% in all four countries. However, progress is not swift enough; we estimate that about one in 10 women in South Asia has a low stature and one in five has a low BMI. This is of immense concern because low maternal stature and BMI have been shown to predict child stunting in South Asia (Kim, Mejia‐Guevara, Corsi, Aguayo, & Subramanian, 2017), in addition to other adverse maternal and newborn outcomes. Furthermore, the region is home to over one third of the world's anaemic women, and no country in the region is on track to meet the World Health Assembly target to halve anaemia in women of reproductive age by 2025 (Development Initiatives, 2017).

Given the close links between maternal and child nutrition, efforts to improve the nutritional status of women are critical to attaining the global nutrition targets of the Sustainable Development Goals and World Health Assembly and in unleashing the developmental potential of children and the economic development of South Asian nations (Aguayo & Menon, 2016; Vir, 2016; Development Initiatives, 2017). Long‐running interventions such as IFA supplementation are reaching far too few pregnant women in the region, although there is considerable variation both between and within countries. The most recent demographic and health surveys (DHS) show that the proportion of women receiving IFA supplements for at least 90 days during their most recent pregnancy ranges from 7% in Afghanistan (DHS 2015) to 71% in Nepal (DHS 2016), whereas interstate variation in India ranges from 4% in Nagaland to 82% in Lakshadweep (National Family Health Survey 2015–2016).

In light of the poor maternal nutrition indicators in South Asia and poor coverage of evidence‐based interventions, this review was conducted to (a) identify drivers and barriers to the coverage of maternal nutrition interventions and (b) examine the evidence on the effectiveness of programme approaches and actions to improve coverage.

## 2 METHODS

### 2.1 Eligibility criteria

Eligibility criteria for the studies included the study location (Afghanistan, Bangladesh, Bhutan, India, Maldives, Nepal, Pakistan and Sri Lanka), year of publication (2000–2017, to capture recent findings that are likely to remain applicable in the present‐day), participant type (pregnant women), study design, at least one maternal nutrition intervention, and at least one outcome measure of interest. Eligible study designs included individual and cluster randomized controlled trials (RCTs), quasi‐randomized with either individual or cluster randomization and non‐RCTs, controlled before and after studies (cohort or cross‐sectional), interrupted time series, and historically controlled studies. Qualitative studies that reported on barriers and facilitators for intervention coverage were also included. Eligible maternal nutrition interventions included micronutrient supplementation (iron and folic acid [IFA], calcium, and vitamin A) and dietary counselling to improve dietary intake. Supplementation with multiple micronutrients was not included as it is not a recommended nutrition intervention in the WHO (2016) guidelines on ANC. The outcome measures of interest included (a) the receipt of the maternal nutrition intervention, as defined by the proportion of women who received the maternal nutrition intervention during pregnancy and (b) the consumption of micronutrient supplements, as defined by the proportion of women who consumed any or a specific number of micronutrient supplements during pregnancy.

### 2.2 Search strategy

Research articles and grey literature published between January 2000 and December 2017 were identified from 13 electronic bibliographic databases and grey literature sources (Cochrane Central Register of Studies, PubMed, MEDLINE, IBECS [English], CINAHL [EBSCO], Popline, UNSCN, Google Scholar, Index Medicus for South‐East Asia Region, Virtual Health Sciences Library, e‐Library of Evidence for Nutrition Actions, Global database on the Implementation of Nutrition Action, and Nutrition Landscape Information System). The search strategy was based on a location search term AND/OR one population group search term AND/OR one maternal nutrition search term AND/OR one study type and was adapted to each of the 13 database requirements. Boolean operators and Medical Subject Headings were applied (Table 1). No language restriction was applied.

**Table 1:** Search terms by criteria

Criteria	Search terms
Country	Afghanistan OR Bangladesh OR Bhutan OR India OR Nepal OR Maldives OR Pakistan OR Sri Lanka OR Asia OR South Asia
Woman	Pregnant OR pregnant woman OR pregnant women OR pregnancy
Maternal nutrition	(maternal nutrition) OR (antenatal care) OR ANC OR vitamin A OR micronutrient OR (maternal health) OR (community health) OR (health access) OR anaemia OR anaemic OR (community health) OR (health access) OR coverage OR (nutrition counselling) OR counselling OR calcium OR iron
Study type	RCT OR (randomized controlled trial) OR (randomized controlled trial) OR (randomized control trial) OR (randomized control trial) OR (quasi randomized) OR (quasi randomized) OR (nonrandomized controlled trial) OR (nonrandomized controlled trial) OR (nonrandomized control trial) OR (nonrandomized control trial) OR (historically controlled stud*) OR (interrupted time series) OR (before and after study) OR (systematic review) OR (cohort study) OR (cross‐sectional study) OR (longitudinal study) OR (cross‐sequential study) OR (meta‐analysis) OR (literature review) OR (qualitative) OR (evaluation)

*Note*. MESH terms used (in PubMed): maternal nutritional physiological phenomena, prenatal care, vitamin A, maternal health, public health, health, counselling, nutritional status, randomized controlled trials.

In addition, grey literature, including programme evaluations and unpublished literature, was sourced from the organizational websites of 14 agencies, academic institutions, and technical bodies using an adapted search strategy for individual websites (Table S1) and was solicited from development partners working in the South Asia region.

### 2.3 Study selection

One researcher performed the database and website searches using the aforementioned criteria and search strategy. All titles and abstracts of study reports were screened for inclusion by the first author, and a randomly selected sample was checked by the second author. The full text of potentially eligible studies was retrieved for screening against the eligibility criteria, and the selection of the final set of studies was confirmed by the second and third authors.

### 2.4 Data extraction and synthesis

The selected studies were examined separately by the first three authors. A standardized data extraction template was used to extract data including the title, authors, publication year, country, study design, intervention and comparison, target population, sample size, outcome measure, and results. The study aims and recommendations were also recorded. Data were synthesized by type of maternal nutrition intervention and type of barrier to coverage of or adherence with the intervention. Barriers were identified at three levels: individual, household, and health service delivery.

### 2.5 Quality assessment

The quality of evidence was assessed using the Grading of Recommendations, Assessment, Development, and Evaluation criteria based on the eight known assessment criteria (Atkins et al., 2004; Walker, Fischer‐Walker, Bryce, Bahl, & Cousens, 2010). Two authors reviewed the evidence and made a judgement about quality.

### 2.6 Equity assessment

We aimed to assess equity by using the PROGRESS framework (place of residence, race/ethnicity, occupation, gender, religion, education, socio‐economic status, and social capital) for the included studies (O'Neill et al., 2014).

## 3 RESULTS

### 3.1 Characteristics of the included studies

**Figure 1:**
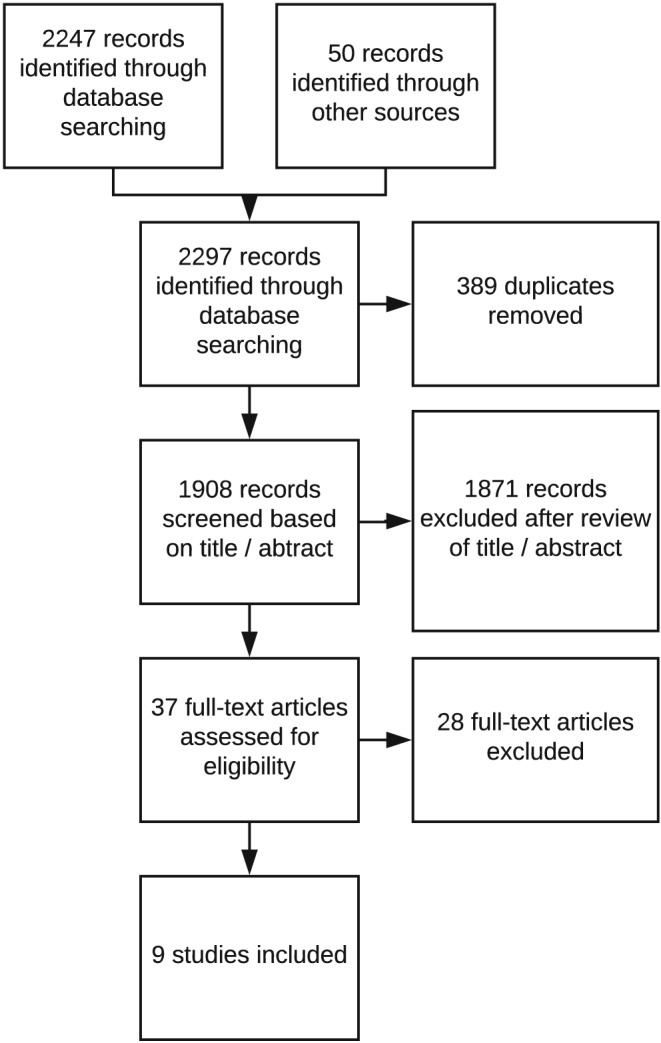
Study selection process for the review

The searches identified 2,247 citations (Figure 1). After removing duplicate citations and screening titles and abstracts, 37 articles were considered potentially eligible. After a full‐text review, 28 studies were excluded because the study participants were not pregnant women and/or the study did not examine predictors or the coverage of a WHO‐recommended maternal nutrition intervention. The remaining nine studies met the inclusion criteria and included two studies conducted in Bangladesh (Nguyen et al., 2017a; Nguyen et al., 2017b), five in India (Balakrishnan et al., 2016; Ghanekar, Kanani, & Patel, 2002; Prinja et al., 2017; Shivalli, Srivastava, & Singh, 2015; Wendt et al., 2015), one in Nepal (Sharma et al., 2016), and one in Pakistan (Nisar, Dibley, & Mir, 2014). These eligible studies included one cluster RCT (Nguyen, Kim, et al., 2017a), four quasi‐experimental studies (Balakrishnan et al., 2016; Prinja et al., 2017; Sharma et al., 2016; Shivalli et al., 2015), three cross‐sectional studies (Nguyen, Sanghvi, et al., 2017b; Nisar et al., 2014; Wendt et al., 2015), and one qualitative study (Ghanekar et al., 2002). All the nine studies examined the receipt and/or consumption of IFA supplements during pregnancy, two studies examined the receipt and/or consumption of calcium supplements (Nguyen, Kim, et al., 2017a; Nguyen, Sanghvi, et al., 2017b), and one study examined the receipt of information on maternal diet (Nguyen, Kim, et al., 2017a). There were no studies that reported the coverage of other maternal nutrition interventions. A summary of the included studies is provided in Table 2 for cross‐sectional studies and Table 3 for all intervention studies.

**Table 2:** Summary of included cross‐sectional studies examining predictors of the receipt or consumption of supplements

Source and country	Sample size (women)	Survey data source and context	Significant predictors
Nguyen, Sanghvi, et al. (2017b) Bangladesh	2,600	Data source for analysis was a baseline household survey conducted in 2015 as part of an evaluation to test feasibility and impacts of integrating intensified maternal nutrition interventions into the existing maternal newborn and child health (MNCH) programme. Survey conducted in 20 rural subdistricts from four districts where the MNCH programme had been in place for more than 5 years.	Consumption: Maternal knowledge was strongly associated with higher consumption of IFA (β = 32.5, 95% CI [19.5, 45.6]) and calcium supplements (β = 31.9, 95% CI [20.9, 43.0]). Compared with women with low knowledge, those with medium knowledge consumed 19 more IFA supplements (*p* < 0.01) and 23 more calcium supplements (*p* < 0.001), and those with high knowledge consumed 31 more IFA supplements (*p* < 0.001) and 30 more calcium supplements (*p* < 0.001). Women who reported a high level of husband's support were more likely to consume IFA supplements (β = 25.0, 95% CI [18.0, 32.1]) and calcium (β = 26.6, 95% CI [19.4, 33.7]) supplements compared with those with low support. Early and more prenatal care visits and receipt of free supplements was attributed to the consumption of an additional 46 IFA and 53 calcium supplements.
Wendt et al., 2015 India	7,765	Data source was the third round of the 2007–2008 district level household survey in the state of Bihar, where IFA supplements are distributed to women through antenatal care at health subcenter facilities.	Receipt: 37% of women received any IFA supplements during their last pregnancy, of which 24% consumed IFA supplements for 90 or more days. Women were more likely to receive any IFA supplements when they received additional ANC services and counselling, and attended ANC earlier and more frequently. Significant interactions were found between ANC quality factors relating to counselling (OR: 0.37, 95% CI [0.25, 0.56]) and between ANC services and ANC timing and frequency (OR: 0.68, 95% CI [0.56, 0.82]). Consumption: Women were more likely to consume IFA supplements for at least 90 days if they attended at least four ANC check‐ups and enrolled early (OR: 3.4, 95% CI [2.52, 4.59]), and if they were in the richest wealth quintile or had had at least 9 years of education. IFA supply at the HSC was significantly associated with IFA consumption (OR: 1.37, 95% CI [1.04, 1.82]).
Nisar et al., 2014 Pakistan	6,266	Data source was the end line survey conducted in 14 project districts, where the family advancement for life and health project was implemented to increase the demand and utilization of reproductive health services.	Consumption: Women were less likely to consume any IFA supplements if they were aged at least 45 years (OR: 1.97, 95% CI [1.07, 3.62]), had no education (OR: 2.36, 95% CI [1.65, 3.37]), husband had no education (OR: 1.58, 95% CI [1.27–1.97]), the household belonged to the lowest wealth index quartile (OR: 1.47, 95% CI [1.11, 1.93]) and had no use of ANC services (OR: 13.39, CI [10.70, 16.75])

**Table 3:** Summary of the included intervention studies (source, country, study design, sample size, intervention description, and results)

Source and country	Study design	Sample size (women)	Intervention description, including barrier type, intervention group (IG) and comparison group (CG)	Results by outcome indicator
Balakrishnan et al., 2016 India	Quasi‐experimental	16,000	Barriers type: Individual, service delivery IG: Front‐line health workers were trained on the provision of maternal and child health care and on the utilization of a mobile health (mhealth) technology to support the continuum of maternal and child care services. The mhealth application included a home visit planner, built‐in scheduler, checklists and videos to help front‐line workers perform activities, improve interpersonal communication, and collect data. CG‐A: Routine health services—previous year in same district CG‐B: Routine health services—nonintervention districts in the same state	% women who received more than 90 IFA supplements during pregnancy**:** Higher in IG (61.6%, 95% CI [44.3, 68.4]) than CG‐A (50.2%) and CG‐B (49.0%), but no significant difference.
Ghanekar et al., 2002 India	Qualitative	36	Barrier type: Individual, household IG: An ethnographic decision model was used to identify barriers to the consumption of purchase of IFA and compliance with IFA supplementation. Fortnightly home visits were made to pregnant women and family members to promote and counsel on IFA purchase and compliance, and continued until end of pregnancy. CG: Not applicable	% of target dose of IFA supplements consumed by pregnant women: 95% of the women consuming over 90% of the required dose.
Nguyen et al. 2017a Bangladesh	Cluster RCT	300	Barrier type: Individual, household, service delivery IG: An intensified, nutrition‐focused package of interventions was integrated into an existing maternal, neonatal, and child health (MNCH) programme with the goal of improving maternal diet quality, micronutrient intakes, and breastfeeding practices. It included greater specificity of interpersonal counselling, free IFA and calcium supplements, pregnancy weight‐gain monitoring, explicit engagement of husbands, and community mobilization activities. CG: Standard MNCH (antenatal care with standard nutrition counselling)	% women who received only free IFA supplements during pregnancy: Increase between baseline and end line greater in IG (44.8% to 96.5%) than CG (53.3% to 42.4%). Difference‐in‐difference effect estimate 62.6 pp, *p* < 0.001. % women who received only free calcium supplements during pregnancy: Increase between baseline and end line greater in IG (31.5% to 96.3%) than CG (42.4% to 34.9%). Difference‐in‐difference effect estimate 72.3 pp, *p* < 0.001. % women who consumed IFA supplements during pregnancy: Increase between baseline and end line greater in IG than CG: Difference‐in‐difference effect estimate 9.8 pp (*p* < 0.001). % women who consumed calcium supplements during pregnancy: Increase between baseline and end line greater in IG than CG: Difference‐in‐difference effect estimate 12.8 pp (*p* < 0.001). Number of IFA and calcium supplements consumed: Significant effects on the number of IFA and calcium supplements consumed (effects: 46 and 50 supplements, respectively), *p* < 0.001. % women received information to eat five varieties of food during pregnancy: Increase between baseline and end line greater in IG (29.5% to 82.3%) than CG (36.6% to 22.9%). Difference‐in‐difference effect estimate 66.5 pp, *p* < 0.001. % women received information to additional amounts of food during pregnancy: No significant change between baseline and end line in IG or than CG.
Prinja et al., 2017 India	Quasi‐experimental	2,444	Barrier type: Individual, service delivery IG: A mHealth application was integrated into an MNCH programme for use as a job aid by community health workers for registering pregnant women and for providing real‐time guidance. The application provided guidance on key counselling points, decision support and simple referral mechanisms for various maternal and child health conditions. CG: Two other blocks from the same district, matched for Antenatal care and institutional deliveries, where mHealth application was not introduced	% women who consumed more than 100 IFA supplements during pregnancy: For matched analysis, decrease between baseline and end line in IG (2.0% to 1.0%) significantly less than CG (14.1% to 0.4%), difference‐in‐difference 12.7 pp (*p* < 0.001)
Sharma et al., 2016 Nepal	Quasi‐experimental	1,236	Barrier type: Individual, household, service delivery IG: A community‐based maternal health promotion intervention was implemented, involving women's health groups and participatory activities with visual cards and role‐playing to improve uptake of maternal health and nutrition services. CG: One district with similar socio‐economic conditions in which no community‐based maternal health promotion interventions were delivered.	% women who consumed IFA supplements during pregnancy: Significant increase between baseline and end line in IG (86.6% to 96.0%, *p* < 0.001) but not CG (76.4% to 79.3%).
Shivalli et al., 2015India	Quasi‐experimental	86	Barrier type: Individual, household IG: Trials for improved practices (TIPs) conducted to enhance dietary intake and IFA consumption during pregnancy. It involved an assessment visit (interviews of pregnant women and her family members and observations to ascertain barriers to IFA consumption), a negotiation visit (use of communication and counselling guide with appropriate messages and pictures and a home‐based reminder tool to encourage IFA consumption) and an evaluation visit (to assess whether the pregnant woman was able consume adequate IFA supplements). CG: Two villages in which TIPs was not conducted.	% of women who consumed IFA supplements for at least 100 days during pregnancy: 85% of the women in TIPs group vs. 38% in comparison group.

### 3.2 Barriers and enablers to the receipt and consumption of supplements

**Figure 2:**
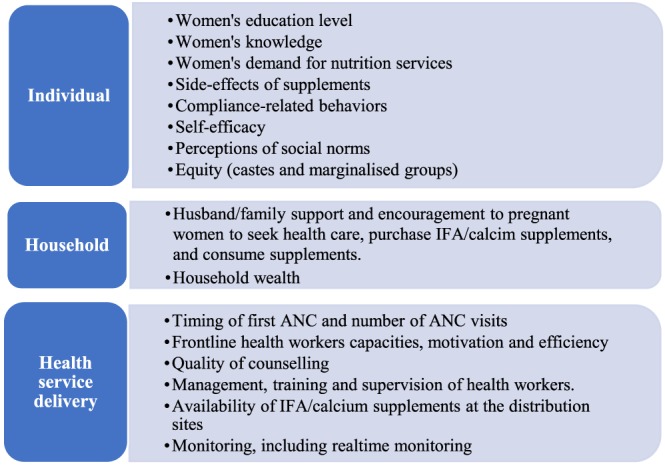
Barriers/enablers to the receipt and consumption of iron folic acid (IFA) and calcium supplements at individual, household, and service delivery level

Figure 2 shows barriers (or enablers) to the receipt and consumption of IFA and calcium supplements derived from the analysis of predictors for the two cross‐sectional studies and the barriers that were addressed by the intervention studies listed in Table 3. Barriers are identified at individual/woman, household, and health service delivery levels.

Individual factors include women's education level, women's knowledge, demand for services, compliance‐related behaviours, self‐efficacy to follow recommended practices, social norms, and cultural beliefs (Ghanekar et al., 2002; Nisar et al., 2014; Shivalli et al., 2015; Wendt et al., 2015; Balakrishnan et al., 2016; Sharma et al., 2016; Nguyen, Kim, et al., 2017a; Nguyen, Sanghvi, et al., 2017b; Prinja et al., 2017).

Household factors include husband/family support and encouragement to the pregnant woman to seek health care and to purchase and consume supplements, husband's education, and household wealth (Ghanekar et al., 2002; Nisar et al., 2014; Shivalli et al., 2015; Wendt et al., 2015; Sharma et al., 2016; Nguyen, Kim, et al., 2017a).

Health service delivery factors include the timing of the pregnant woman's first ANC visit, the number of ANC visits, health worker capacities, quality of dietary counselling, supply of IFA/calcium supplements to distribution sites, and provision of free supplements **(**Nisar et al., 2014; Wendt et al., 2015; Balakrishnan et al., 2016; Sharma et al., 2016; Nguyen, Kim, et al., 2017a; Prinja et al., 2017).

Only two intervention studies, one on the nutrition‐focused Maternal Neonatal Child Health (MNCH) in Bangladesh (Nguyen, Kim, et al., 2017a) and the other on community‐based maternal health promotion intervention in Nepal (Sharma et al., 2016) aimed to address barriers at all three levels; all other studies addressed barriers at two levels.

### 3.3 Effectiveness of programme approaches to increase the coverage of maternal nutrition interventions

#### 3.3.1 Quality of evidence

The overall quality of evidence was assessed for five intervention studies using Grading of Recommendations, Assessment, Development, and Evaluation criteria for the receipt or consumption of IFA supplements, the most common outcome indicators, and was found to be low to moderate quality; the qualitative study, Ghanekar et al. (2002), was excluded from this assessment. The five studies (four quasi‐experimental and one cluster RCT) included 20,066 participants. Several study design biases limited the quality of the evidence: In Balakrishnan et al. (2016), there was a potential performance bias as the data from the intervention areas were compared with the data from the rest of Bihar and historical data from previous year; in Prinja et al. (2017) and Sharma et al. (2016), there was potential selection due to the lack of randomization; and in Shivalli et al. (2015), the small sample size was calculated using coverage parameters that were very different from the results. There was heterogeneity in the study designs and methods; only one study used an RCT and others were quasi‐experimental with various designs. Clinical heterogeneity was noted due to the difference in types of interventions and outcomes used (Table S2). In terms of consistency, effects on the IFA coverage indicator were positive, inconclusive, or negative (Table 3). There was additionally imprecision in the effect size due to large confidence interval (Balakrishnan et al., 2016). As there was only one RCT included in this review (Nguyen, Kim, et al., 2017a), we downgraded a starting rating of “high quality” evidence (for RCT) by two levels for very serious concerns about risk of bias, clinical and methodological heterogeneity, inconsistency, and imprecisions.

There was variation across the seven interventions studies in the dose of iron, folic acid, and calcium in supplements and the definition of the outcome indicators on IFA and calcium supplementation with respect to the recall period (previous month of pregnancy or the entire duration of pregnancy) and the number of supplements received or consumed (Table S2). This variation renders it difficult to draw coverage comparisons across the studies.

#### 3.3.2 Equity

Although most studies reported the socio‐economic status and demographic information of the women at baseline, only Sharma et al. (2016) disaggregated the outcome variables by wealth and education and showed that having higher education and being wealthier were significant factors in the consumption of IFA supplements during pregnancy. We were therefore not able to assess equity for the outcomes on the receipt or consumption of IFA supplements.

#### 3.3.3 Receipt of supplements

The only study that reported a positive impact on the receipt of supplements was the nutrition‐focused MNCH study in Bangladesh, which reported significant effects on the receipt of free IFA supplements and free calcium supplements by pregnant women in Bangladesh (Nguyen, Kim, et al., 2017a). In this programme, nutrition‐focused interpersonal counselling, community mobilization, and the distribution of free IFA and calcium supplements to women at home were integrated into an existing MNCH programme and compared with standard ANC services and dietary counselling. The programme approaches were informed by formative research that indicated that early initiation of supplementation, a higher number of ANC visits (more than four visits versus four visits and less), and the provision of free supplements were potential drivers of the receipt of supplements (Nguyen, Sanghvi, et al., 2017b). Health workers and volunteers were highly trained and closely supervised, and health volunteers received performance‐based incentives according to the number of new pregnant women reached, home visits made, and pregnant women practicing new behaviours. Close monitoring by the district manager and an independent team of five monitors was conducted to track the performance of front‐line workers. The proportion of pregnant women receiving free IFA or calcium supplements more than doubled between baseline and end line in the intervention group but decreased by more than 7 percentage points (pp) in the comparison group (difference‐in‐difference effect size (DDE) 62.6 pp for IFA and 73.3 pp for calcium, *p* < 0.001). Nguyen, Kim, et al. (2017a) highlighted that the study was carried out in the context of a well‐functioning and robust MNCH programme and that effects may be less in the absence of these conditions.

Balakrishnan et al. (2016) explored the effect of a mobile health (mhealth) application in strengthening the delivery of health and nutrition services by front‐line workers to pregnant women in Bihar, India, and reported no significant effect on the percentage of women who received more than 90 IFA supplements. Balakrishnan et al. (2016) noted that there was a significant improvement in the coverage of other health services to pregnant women in the intervention group. They indicated that the lack of impact on IFA supplement receipt was due to unresolved barriers, including a shortage of IFA supplements to distribution sites in the intervention area and the fact that the front‐line workers using the mhealth application were not responsible for the supply of IFA supplements to pregnant women.

#### 3.3.4 Consumption of supplements

Studies reporting a positive impact on the consumption of supplements included Nguyen, Kim, et al. (2017a) for consumption of any IFA/calcium supplements and the number of IFA/calcium supplements consumed in Bangladesh; Sharma et al. (2016) for consumption of any IFA supplements in Nepal; and Shivalli et al. (2015) for consumption of IFA supplements for at least 100 days in India (Table 3).

The nutrition‐focused MNCH programme in Bangladesh (Nguyen, Kim, et al., 2017a) included a number of initiatives to increase the consumption of supplements by pregnant women in the intervention areas. Women were counselled during home visits on the benefits of IFA and calcium supplements, consequences of IFA or calcium deficiency, doses and duration of IFA and calcium supplementation that should be followed, side effects of supplements and ways to minimize them, and foods that may inhibit their absorption. Husbands and other family members were engaged to ensure women took sufficient supplements during home visits and community mobilization events such as husband forums. In addition, women were requested to preserve empty strips of supplements to monitor how many they took. The increase between baseline and end line in the proportion of women who reported to consume supplements during pregnancy was greater in the intervention group than comparison group for both IFA (DDE 9.8 pp, *p* < 0.001) and calcium (DDE 12.8 pp, *p* < 0.001). Significant effects were also observed for the number of supplements consumed for both IFA (additional 46 supplements) and calcium (additional 50 supplements) in the intervention group (*p* < 0.001). The authors attribute these results to the high priority given to ensuring adequate supplies of free supplements to women at home, the high quality of training and supervision of front‐line workers, the performance‐based incentives for community volunteers, and high quality of counselling involving home visits and husbands.

In Nepal, a participatory community‐based maternal health promotion intervention that focused on community groups to improve the uptake of maternal health services significantly increased the proportion of women who reported to consume IFA supplements during pregnancy between baseline and end line by 9.4 pp (*p* < 0.001); there was no significant increase in the comparison group (Sharma et al., 2016). The community groups comprised women aged 15–49 years with at least one child under the age of 2 years, some mixed with mothers‐in‐law groups, and separate men's group. The interventions consisted of 24 one‐hr health promotion sessions covering a range of issues related to health care during pregnancy, delivery, and the postnatal period, including the importance of IFA supplementation. Participatory activities with visual cards and role playing were conducted, and efforts were made to encourage group attendance by empowering lower castes to attend and providing small incentives to women on completion of four ANC visits. The results suggest that community‐based health promotion groups involving mothers‐in‐law and husbands as well as pregnant women can improve the consumption of IFA supplements during pregnancy.

Shivalli et al. (2015) examined the effectiveness of trials for improved practices to increase the consumption of IFA supplements during pregnancy in Varanasi, India. The trials for improved practices involved an assessment home visit to ascertain the perceptions and practices related to IFA consumption from pregnant women and her family members, followed by a negotiation home visit based on the findings of the assessment, in which pregnant women were asked to select and try individually tailored practices and were given home‐based reminder tools. The proportion of women who reported to consume at least 100 IFA supplements during pregnancy was 85% in the intervention group, compared with only 38% in the comparison group. The authors reported that pregnant women in the intervention group appreciated the counselling, reminder materials, and family support, which together helped to motivate and sustain IFA consumption throughout pregnancy.

A study conducted in Vadadara, India (Ghanekar et al., 2002), had no baseline or comparison groups, and so it was not possible to determine the effect of the programme interventions on IFA supplement consumption with certainty. However, the study offers important insights into approaches to address barriers to IFA supplement consumption. In this study, qualitative information on factors influencing IFA supplement consumption was obtained from poor slum residents, pregnant women and their husbands, or mothers‐in‐law through one or more in‐depth interviews. This information was used to develop ethnographic decision models that depict the decision‐making pathways to the consumption or nonconsumption of IFA (family member's approval to take supplements, experiences of benefits or side effects, forgetfulness to take supplements, and/or family member reminder/encouragement to take supplements, etc.). The authors reported that regular counselling, using a flip‐chart based on the findings of the qualitative research, helped to motivate women to continue taking supplements and cope with any side effects and resulted in over 95% of women consuming over 90% of the required number of supplements during the previous month. Family support, together with positive experiences of the benefits of IFA supplementation and regular home visits by front‐line workers, was key in motivating women to consume IFA supplements. These results are noteworthy, given the low consumption of IFA in India around the time of the study: Only 23% women consumed iron supplements for at least 90 days during their most recent pregnancy, according to the National Family Health Survey, which was conducted in 2005–2006. The study demonstrates the contribution of qualitative ethnographic data to developing appropriate counselling materials and strategies.

Prinja et al. (2017) examined the effect of a mhealth application to increase the capacity of front‐line workers in Uttar Pradesh, India, to deliver quality counselling to pregnant women, defined as complete and accurate information, and to generate demand for ANC services, including IFA supplementation. The study found a decrease in the percentage of women who reported to consume more than 100 IFA tablets during pregnancy between baseline and end line in both the intervention and comparison groups. Although the decrease was smaller in the intervention group (from 2.0% to 1.0%) than the comparison group (from 14.1% to 0.4%), the baseline value in the comparison group was considerably higher, and the study did not provide conclusive evidence that the programme intervention was effective in improving the consumption of IFA supplements. It is likely that the mhealth application alone was insufficient to improve consumption of IFA supplements.

#### 3.3.5 Counselling on maternal diet

The nutrition‐focused MNCH programme in Bangladesh (Nguyen, Kim, et al., 2017a) significantly increased the proportion of pregnant women who received information on eating five food groups during pregnancy but not the proportion who received information on consuming additional amounts of food during pregnancy. Women in the intervention group consumed 1.6 more food groups had higher increases in the proportion consuming high‐nutritional value foods such as pulses, dairy, meat, and eggs and consumed greater quantities of food than women in the comparison group. These results were achieved through a mix of strategies aimed at influencing behaviour change, including monthly home visits by health workers, involving demonstrations of a specific diet plan (both quantity and quality), and engagement of other family members to improve the dietary intake of pregnant women through home visits and husband forums at the community level.

## 4 DISCUSSION

This systematic review examines evidence from nine studies published between 2000 and 2017 on the predictors of the coverage of maternal nutrition interventions and effectiveness of programme actions to improve coverage in South Asian countries. The studies indicate that a range of barriers acting at the individual (maternal), household, and health service delivery level affects whether a woman receives maternal nutrition interventions and, in the case micronutrient supplementation, whether she consumes the supplements. Programme approaches that were effective in increasing the receipt of services and/or consumption of supplements used a combination of actions to address barriers at multiple levels.

Predictors of the receipt and consumption of IFA and calcium supplements in Bangladesh, India, and Pakistan included early and more frequent ANC visits, higher maternal education, higher paternal education, higher maternal knowledge, increased household wealth, access to counselling services, and a higher level of support from husbands. These enablers confirm previous findings on IFA supplementation that point to the role of women's knowledge and empowerment, household resources, women's access health services, and the quality of health services in determining whether women receive and consume supplements and adopt positive dietary practices (Kavle & Landry, 2017; Kavle & Landry, 2018; Siekmans, Roche, Kung'u, Desrochers, & De‐Regil, 2017; Sununtnasuk, D'Agostino, & Fiedler, 2016).

All programmes that delivered information and counselling to women and their family members, often in combination with other programme actions, significantly improved the receipt and consumption of IFA and calcium supplements and the receipt of information on a diverse diet during pregnancy (Ghanekar et al., 2002; Nguyen, Kim, et al., 2017a; Sharma et al., 2016; Shivalli et al., 2015). These programmes had several common features. First, they used formative research and ethnography to adapt the design of programme actions to the context‐relevant barriers faced by women in accessing services or adopting recommended nutrition behaviours. This included the design of locally relevant information and counselling materials. Second, they engaged with and enlisted the support of influential family members, including husbands and mothers‐in‐law, to ensure pregnant women accessed services and adopted positive behaviours, thereby countering women's low decision‐making authority. Third, they introduced home visits and community forums to increase the access of pregnant women, influential family members, and other community leaders to interventions. Fourth, they developed the capacity of front‐line workers to provide information and counselling to women, family members, and other community members, and in Bangladesh (Nguyen, Kim, et al., 2017a), added mechanisms to supervise, monitor, and motivate their performance.

The two programmes that examined the effectiveness of mhealth applications to improve service delivery by front‐line workers were not effective in increasing the receipt or consumption of IFA supplies (Balakrishnan et al., 2016; Prinja et al., 2017). It is likely that mhealth approaches alone will not be effective if other barriers to receipt or consumption of IFA, such as a lack of IFA supplies, are not addressed.

These findings confirm previous research that highlight the value of community‐based intervention packages for improving maternal care and neonatal outcomes (Lassi & Bhutta, 2015). A recent review of global evidence found that community‐based distribution of IFA supplements provides an effective platform to improve knowledge about anaemia, provide counselling to improve compliance, and should be coupled with actions to ensure the consistent supply of supplements at facility and community levels to improve both access and coverage (Kavle & Landry, 2018). In addition, a separate review highlighted the importance of tailored, culturally appropriate nutrition education and counselling during pregnancy (Kavle & Landry, 2018).

There are considerable gaps in the body of evidence on the effectiveness of programme approaches to improve the coverage of maternal nutrition interventions in South Asia. Most studies examined only IFA supplementation, and only one examined calcium supplementation and access to information on dietary intake during pregnancy, even though all countries in the region are delivering multiple nutrition interventions to women during pregnancy. We found no published evidence that met the review criteria from Afghanistan, Bhutan, Maldives, and Sri Lanka, only one study in Nepal and Pakistan, and two in Bangladesh. Most of the studies reviewed lacked detailed programme impact pathways making it challenging to assess which approaches, actions, or processes contributed to the changes in receipt or consumption of the interventions. Design issues and nonstandardized use of indicators among the included studies meant that it was not possible to compare the results between studies. The quality of the evidence on IFA supplementation coverage (receipt and consumption) was assessed as low to moderate due to risk of bias, clinical and methodological heterogeneity, inconsistency, and imprecision.

Greater attention is needed in all country settings in South Asia to resourcing and conducting formative and implementation research to better understand the pathways, enablers, and barriers to improving the access to all maternal nutrition interventions recommended by the WHO (2016), including the quality of counselling, and adoption of positive maternal nutrition behaviours. In addition, analysis on the cost‐effectiveness of approaches to integrate maternal nutrition interventions into ANC and MNCH services at facility and community levels and the cost of scale‐up would provide valuable evidence to guide programme prioritization and allocation decisions.

## 5 CONCLUSION

Pregnant women require quality nutritional care throughout pregnancy to safeguard their own health and the growth, development, and health of their children. This systematic review of recent evidence from three countries in South Asia indicates that a range of barriers acting at the individual (maternal), household, and health service delivery level affects whether a woman receives maternal nutrition interventions and, in the case micronutrient supplementation, whether she consumes the supplements. This systematic review highlights that actions to reach pregnant women with IFA and calcium supplements in their homes and communities, combined with information and counselling, can improve the access to services and consumption of supplements, particularly when these approaches are based on formative research, engage influential family members, increase the capacities, supervision and motivation of front‐line workers, and ensure that the supply of supplements is not interrupted. These findings are likely to apply in other low‐ and middle‐income countries where women experience similar barriers to accessing and utilizing maternal nutrition services during pregnancy. The evidence base on what works to improve the coverage of maternal nutrition interventions in South Asia is very small, and greater investments in implementation research and evaluations are needed to enhance the understanding of the barriers, enablers, and pathways to increasing coverage across programmes and diverse contexts in the region.

## CONFLICTS OF INTEREST

The authors declare that they have no conflicts of interest.

## CONTRIBUTIONS

SG, ZM, and HT were involved in the conception, design, and collection of data. SG led the analysis, and SG, ZM, and HT interpreted the data and prepared the manuscript. JH and JBH contributed to the interpretation of the data and to the drafting of the manuscript. All named authors critically reviewed the paper and approved the final version submitted for publication.

## Supporting information

Table S1: List of organizational websites searched.
Table S2: Iron folic acid (IFA) and calcium dose, adherence and outcome indicators by study
