# Supplementary material for: Effectiveness of programme approaches to improve the coverage of maternal nutrition interventions in South Asia
Source: Matern Child Nutr. 2018 Nov 29;14(Suppl 4):e12699. doi: 10.1111/mcn.12699 (PMC6519063; doi:10.1111/mcn.12699)
Supplement: Supplementary file 1 — Table S1: List of organizational websites searched. Table S2: Iron folic acid (IFA) and calcium dose, adherence and outcome indicators by study [file MCN-14-e12699-s001.docx]

**Supplementary Table 1: List of organizational websites searched.**

| **Organization** | **Website** |
| --- | --- |
| Alive and Thrive | [**htt**p**://aliveandthrive.org/resources-main-page/**](http://aliveandthrive.org/resources-main-page/%20%20) |
| Emergency Nutrition Network | [**http://www.ennonline.net/resources**](http://www.ennonline.net/resources) |
| Food and Nutrition Technical Assistance Project | [**http://www.fantaproject.org/publications**](http://www.fantaproject.org/publications) |
| Global Alliance for Improved Nutrition | [**http://www.gainhealth.org/knowledge-centre/search/**](http://www.gainhealth.org/knowledge-centre/search/) |
| Global Nutrition Cluster | [**http://nutritioncluster.net/tools-and-resources/**](http://nutritioncluster.net/tools-and-resources/) |
| International Centre for Diarrhoeal Disease Research, Bangladesh | [**http://www.icddrb.org/component/search/**](http://www.icddrb.org/component/search/) |
| Nutrition International | [**https://www.nutritionintl.org/knowledge-library/**](https://www.nutritionintl.org/knowledge-library/) |
| United Nations Children's Fund | [**http://www.unicef.org/publications/**](http://www.unicef.org/publications/) |
| United Nations Standing Committee on Nutrition | [**http://www.unscn.org/en/publications/**](http://www.unscn.org/en/publications/) |
| World Bank | [**https://openknowledge.worldbank.org/browse?type=title**](https://openknowledge.worldbank.org/browse?type=title) |
| World Food Programme | [**http://www.wfp.org/publications/list**](http://www.wfp.org/publications/list) |
| World Health Organization (WHO), Department of Child and Adolescent Health and Development | [**http://search.who.int/search**](http://search.who.int/search) |
| UK Department for International Development | [**https://www.gov.uk/government/publications**](https://www.gov.uk/government/publications) |
| United Nations Refugee Agency | [**http://www.unhcr.org/**](http://www.unhcr.org/) |
| United States Agency for International Development | [**http://www.usaid.gov/gsearch/**](http://www.usaid.gov/gsearch/) |

**Supplementary Table 2: Iron folic acid (IFA) and calcium dose, adherence and outcome indicators by study**

| **Source** | **IFA and calcium dose** | **Indicator used** |
| --- | --- | --- |
| Balakrishnan et al. 2016 | Not specified | % women who received more than 90 tablets during last pregnancy |
| Ghanekar et al. 2002 | Not specified | % of target number of IFA supplements consumed by pregnant women |
| Nguyen et al. 2017a | 60 mg iron and 400 μg folic acid  500 mg calcium | % of women who received IFA during last pregnancy  % of women received calcium during last pregnancy  % of women consumed IFA during last pregnancy  % of women consumed calcium during last pregnancy |
| Nguyen et al. 2017b | 60 mg iron and 400 μg folic acid  500 mg calcium | % of women who consumed IFA during last pregnancy  % of women who consumed calcium during last pregnancy  Number of IFA tablets consumed  Number of calcium tablets consumed |
| Prinja et al. 2017 | Not specified | % women who consumed at least 100 IFA tablets during last pregnancy |
| Sharma et al. 2016 | Not specified | % women who consumed IFA during last pregnancy |
| Shivalli et al. 2015 | 100 mg elemental iron and 500 μg folic acid | % women who consumed IFA tablets on at least 100 days during last pregnancy |
| Wendt et al. 2015 | 100 mg elemental iron and 500 μg folic acid | % of women who received IFA during last pregnancy  % women who consumed IFA tablets on at least 90 days during last pregnancy |
